# DNA vaccines: prime time is now

**DOI:** 10.1016/j.coi.2020.01.006

**Published:** 2020-08

**Authors:** Ebony N Gary, David B Weiner

**Affiliations:** The Wistar Institute, Philadelphia, PA, United States

## Abstract

Recently newer synthetic DNA vaccines have been rapidly advanced to clinical study and have demonstrated an impressive degree of immune potency and tolerability. Improvements in DNA delivery over prior needle and syringe approaches include jet delivery, gene gun delivery, among others. Among the most effective of these new delivery methods, advanced electroporation (EP), combined with other advances, induces robust humoral and cellular immunity in both preventative as well as therapeutic studies. Advancements in the design of the DNA inserts include leader sequence changes, RNA and codon optimizations, improved insert designs, increased concentrations of DNA, and skin delivery, appear to complement newer delivery strategies. These advances also provide a framework for the *in vivo* production of synthetic DNA biologics. In this review, we focus on recent studies of synthetic DNA vaccines in the clinic for the prevention or treatment of infectious diseases with a focus on adaptive electroporation for delivery, and briefly summarize novel preclinical data advancing the *in vivo* delivery of DNA-encoded antibody-like biologics.

**Current Opinion in Immunology** 2020, **65**:21–27

This review comes from a themed issue on **Vaccines**

Edited by **Bali Pulendran** and **Rino Rappuoli**

For a complete overview see the Issue and the Editorial

Available online 4th April 2020

**https://doi.org/10.1016/j.coi.2020.01.006**

0952-7915/© 2020 The Author(s). Published by Elsevier Ltd. This is an open access article under the CC BY license (http://creativecommons.org/licenses/by/4.0/).

## Introduction

Vaccines are among the most important medical interventions in human history. We are in an era of unprecedented scientific advance in vaccine technologies. However, the development of new vaccines faces challenges. This is due to multiple factors including the high cost of their development which drives a focus on larger markets, slower than optimal time lines for vaccine advancement to clinical testing, among other complexities. One example is the increased occurrence of emerging and reemerging infections which appear sporadically and could benefit greatly from rapid vaccine interventions. Examples include Lassa, Powasan virus, ZIKA virus (**ZIKV**), Ebola virus (**EBOV**), and the coronaviruses- Middle East respiratory syndrome coronavirus (**MERS-CoV**), severe acute respiratory syndrome coronavirus 1 and 2 (SARS-CoV-1 and SARS-CoV-2)- among many others. An ideal vaccine platform should be simple to deploy, rapid to develop, reproducible, temperature stable, and consistently manufacturable- thus lowering costs and development risks while providing an important new tool. The synthetic DNA (**SynDNA**) platform addresses many of these important goals.

DNA immunogens can be directly designed and optimized from pathogen sequences and synthesized allowing flexibility and speed in preclinical testing with rapid transition to clinical scale up. *In vivo* expression of the constructed sequences facilitates rapid screening and down selection of potential vaccine candidates. Multiple studies have reported that synDNA allows for the generation of cellular and humoral responses against pathogens with impact in challenge model systems. Originally intramuscular (**IM**) inoculation and more recently intradermal (**ID**) delivery using highly concentrated formulations have induced consistent immunity in the clinic. Delivery methods such as jet delivery, gene gun delivery, nanoparticle delivery, and others have demonstrated increased DNA uptake *in vivo* [1^••^].

**Figure 1:**
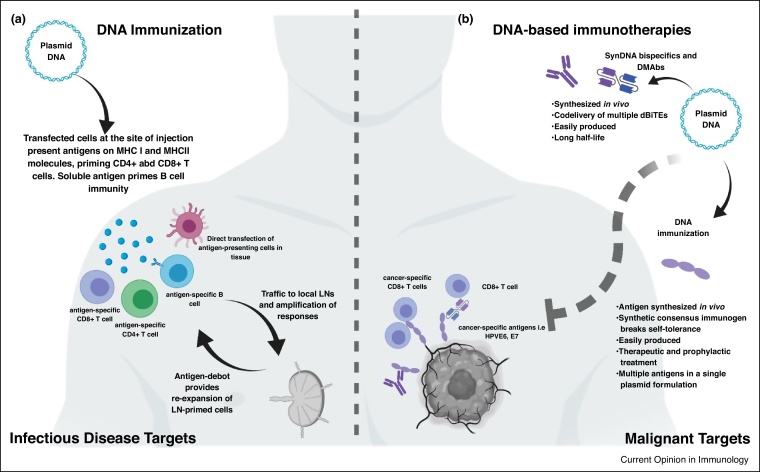
DNA vaccination and immunotherapy. **(a)** DNA-encoded antigens are transcribed, translated, and presented on MHC I and II molecules *in vivo*, promoting robust anti-target immunity. **(b)** The 1000X increase in DNA delivery coupled with highly efficient encoded antigen production allow this local delivery to become a source for production of biologics. Inserts are highly designed to allow for local expression. Multiple publications have now described how DNA-encoded monoclonal antibodies (**DMAb**s), bispecific antibodies, and immunogens can be used to target cancer or infectious diseases.

Adaptive electroporation (**EP**) [2] which controls the energy delivered during *in vivo* EP improves increases transformation efficiency over needle and syringe delivery. Following local injection by needle and syringe plasmid DNA is taken up by a limited number of cells at the site of injection, where the DNA is transcribed into mRNA and translated into antigen intracellularly. Adaptive EP increases the initial uptake of plasmid by local cells approximately 500x [3]. This creates a large antigen bolus to drive a more potent immune response. With adaptive EP most cells in the local field can be transfected [4^••^]. Locally transfected antigen-presenting cells (**APC**s) can directly traffic to the regional lymph node (**LN**) which is critical to initiating the immune response [5,6]. Translated antigen can be shed exogenously and picked up by APCs for cross presentation. Shed exogenous soluble antigen can drain locally to the regional LN and extracellular spaces in the local environment allowing for engagement of B cell immunity. Local tissue becomes a protein factory for presentation of antigen on major histocompatibility complex-1 (MHC I) or MHC II molecules for re-expansion of LN primed CD8^+^ T cells (**cytotoxic T lymphocytes**, or **CTL**s) and CD4^+^ T cells, respectively (Figure 1a). For comprehensive review, see Ref. [4^••^]. Since the 90′s DNA plasmids have been delivered to tens of thousands of patients by multiple routes, in trials studying numerous vaccine antigen targets with a highly consistent safety record supporting its further clinical development [7].

## Clinical review of recent DNA vaccines

**Figure 2:**
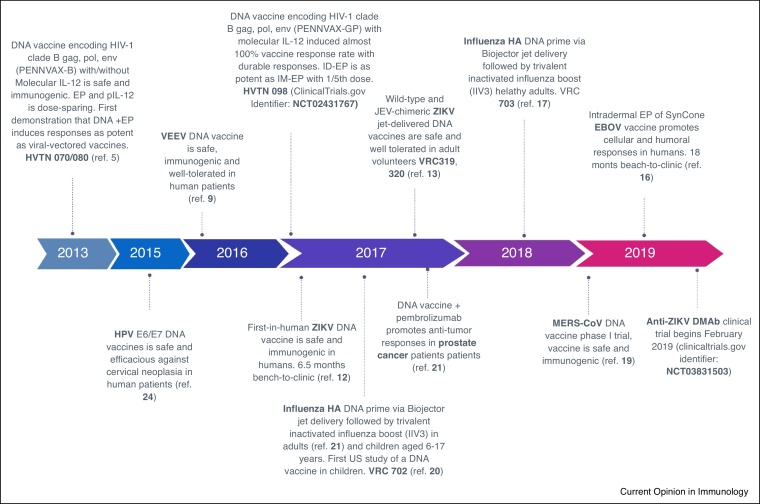
Selected Recent DNA vaccines in the clinic. All the reported constructs were found to be safe and immunogenic in the clinic. Several of these have reported clinical impact or outcomes representing important immune readouts.

Here we discuss recent clinical studies using the SynDNA platform (Figure 2) with a specific focus on DNA vaccines targeting emerging and re-emerging infectious diseases and cancers of infectious etiology.

More than 2 million new human immunodeficiency virus-1 (**HIV-1**) infections occur annually, highlighting the need for an effective prophylactic vaccine. In two multicenter, randomized clinical trials the immunogenicity of synDNA constructs encoding HIV-1 clade B gag, pol, and env proteins (PENNVAX-B DNA vaccine (PV)) alone, or in combination with plasmid-encoded IL-12 was evaluated with (HVTN 080) and without (HVTN 070) intramuscular EP. The vaccination was safe and well-tolerated. Addition of molecular plasmid encoded IL-12 (pIL-12) and EP delivery resulted in a dose sparing effect with immunogenicity superior to vaccines with electroporation, with fewer doses, demonstrating a dose-sparing effect of the combined platform. In the vaccine + IL-12 group with EP delivery, more than 80% of vaccinees had detectable HIV-specific T cell responses after three immunizations [8^••^].

Venezuelan equine encephalitis virus (**VEEV**) is a mosquito-borne alphavirus and is a recognized biosafety threat for humans for which there are no approved vaccines or therapeutics. Hannaman *et al.* studied a DNA vaccine targeting the E3-E2-6K-E1 genes of the VEEV subtype IAB envelope and compared intradermal versus intramuscular electroporation (**EP**) at various doses in a small number of human subjects. In this study high dose intramuscular EP resulted in the development of VEEV neutralizing antibodies in all subjects while intradermal-EP promoted neutralizing antibody at lower levels and in fewer subjects [9]. T cell responses were not reported in this study.

Zika virus (**ZIKV**) is a mosquito-borne infectious disease characterized by fever, rash, conjunctivitis, and malaise. Despite the relatively mild clinical illness, ZIKV infection during pregnancy is associated with severe congenital birth defects such as microcephaly. The first Zika vaccine advanced to the clinic was a synDNA vaccine delivered by adaptive EP [10, 11]. Tebas *et al.* evaluated the safety and immunogenicity of a synthetic DNA vaccine targeting Zika prME, [12^••^]. In this Phase 1 dose-ranging study participants received either 1 or 2 mg of ZIKV DNA immunogens delivered by the ID route and CELLECTRA-EP at 0, 4, and 12 weeks. After the third immunization Zika-specific binding antibodies were detected in all participants at average titers of 1/2000 for the 1 mg dose group or 1/3000 for the higher dose group) 96% of participants developed Zika-neutralizing antibodies detected via U87 neuronal cell assay. Importantly, passive transfer studies where patient serum was given to interferon knockout mice (which are susceptible to ZIKV infection) showed that sera from vaccinated subjects protected mice from lethal pathogenic ZIKV challenge [12^••^]. ZIKV-specific T cells were induced in most subjects. This ZIKV DNA vaccine was moved from research program initiation to the clinic in just 6.5 months highlighting the efficiency and speed with which a synthetic DNA platform can be brought to bear against an emerging or re-emerging pathogen. The Vaccine Research Center (**VRC**) of the national institutes of health reported the results of two ZIKV DNA vaccine trials (VRC 319 and VRC320) evaluating the safety and immunogenicity of DNA plasmids encoding the PrM and E proteins of a ZIKV-Japanese encephalitis virus (JEV) chimera delivered at 4 mg doses either 2x or 3x (VRC 319) or native leader like ZIKV (VRC320) vaccines vaccinated with 4 mg doses immunized three times at different intervals. In VRC 319 GMT average per group was (40–197) and for VRC 320 GMT ranged from (28–430). The vaccines induced neutralizing titers using a reporter assay and showed T cell mediated cytokine release [13] and the authors suggest that the VRC320 vaccine group developed more consistent seroconversion, neutralization, and T cell immunity over the JEV chimeric Zika vaccine.

Ebola virus (**EBOV**) is the causative agent of severe viral hemorrhagic disease in humans and non-human primates. To date, there have been twenty-nine EBOV outbreaks with mortality rates reaching up to 90%. Patel *et al.* reported that rapid *in vivo* protection of mice following a single immunization with a synthetic DNA vaccine encoding consensus EBOV glycoproteins (GP) representative of EBOV outbreak strains spanning the years from 1976 to 2008 against a heterologous mouse-adapted EBOV strain [14]. SynEBOV-GP DNA immunization also resulted in 100% protection of macaques from lethal EBOV challenge [14]. Importantly in these studies determined that a dose-sparing 2-injection regimen delivered via the recently characterized intradermal-EP (CELLECTRA-EP) [15] route was 100% protective against lethal challenge. These responses were persistent up to one year after immunization with robust recall responses observed at this time point [14]. These promising results supported a first in-human (**FIH**) clinical trial. Two EBOV-GP candidates, INO-4201 encoding a SynconEBOV-GP antigen representative of outbreak strains from 1976 to 2008, and INO 4202 encoding Zaire Makona EBOV-GP from a 2014 outbreak, were evaluated alone, together, and in combination with DNA-encoded human interleukin-12 (IL-12) delivered by either the IM or ID routes using Cellectra EP. Both delivery routes induced potent anti-Ebola cellular and humoral immunity. However, the ID delivery route was dose sparing and promoted more rapid seroconversion (100% seroreactivity) after 2 immunizations. The simplicity, consistency, and tolerability of the ID format appears to exhibit important advantages for vaccine development against emerging and re-emerging pathogens [16^••^], and could have a supportive role for the current VSV-ZEBOV vaccine.

The Middle East respiratory syndrome coronavirus (**MERS-CoV**), was first identified in 2012 is responsible for an outbreak of cases which are clustered in the Arabian Peninsula. In 2015 the first spread of MERS to a non-Arabian Peninsula country (Seoul, Korea), generated a major outbreak. Muthumani *et al.* reported on the development of a synthetic DNA vaccine encoding a MERS spike protein which induced potent humoral and cell-mediated immunity in mice, non-human primates, and camels and protected vaccinated macaques from MERS associated pneumonia following virus challenge [17,18]. A phase-I, open-label, single-arm, dose-escalation clinical trial was opened at the Walter-Reed Army Institute for Research Clinical Trials. The vaccine was delivered by intramuscular injection and CELLECTRA-EP. Seroconversion was detected in 95% of patients. MERS-CoV-specific T cell responses were detected in 76% of patients and persisted at 60 days post-final immunization [19^••^].

Influenza viruses which undergo antigenic drift and shift require new vaccine design annually to curb disease spread. Recently the **VRC** of the NIH published two clinical studies of a jet delivered DNA prime, matched, trivalent inactivated virus boost influenza vaccine regimen in healthy adult volunteers, and in children. Compared to inactivated virus prime and boost, DNA prime and virus boost induced trended toward greater humoral responses including hemagglutinin inhibition (HAI) and neutralization [20^••^,^21^]. These findings collectively demonstrate the ability to rapidly apply the synthetic DNA platform in the context of infectious disease or EID, with potent immune performance, as well the excellent tolerability profile of the platform.

## Immunotherapy for viral diseases

Human papilloma viruses (HPV) are oncogenic viruses that infect mucosal surfaces. HPV is responsible for almost 5% of all cancer worldwide [22]. Recombinant HPV particle-based vaccines against specific oncogenic HPVs and two strains of HPV that generate genital warts, have impacted the incidence of HPV infection. However, persons already infected with remain at high risk for a collection of HPV diseases, including cervical cancer, head and neck cancer, anal cancer, vaginal cancer, and others HPV-associated cancers. The first-in-human (**FIH**) clinical trial was advanced to test the hypothesis that a synthetic DNA vaccine encoding HPV immunogens for modified nuclear oncogenes E6 and E7 from HPV types 16 and 18 (two high risk genotypes) would induce CTLs that might impact HPV-induced cervical intraepithelial neoplasia (**CIN**). An initial immunogenicity study [23] reported that the vaccine was highly immunogenic, driving antibodies and CTLs in almost all vaccinated subjects. A follow-up Phase IIb efficacy trail was subsequently reported [24^••^]. The study showed potent induction of immunity including CD8 T cells that migrated to the diseased cervical tissue. Overall 49.5% of women in this study regressed their disease, while 40.2% regressed and cleared their cervical infection. This is the first therapeutic vaccine to show clinical efficacy against grade 2 and 3 CIN [24^••^]. Additional study of this approach for CIN is in progress. This report is also the first treatment efficacy data generated for a DNA approach against a human disease.

The SynDNA platform has the potential to synergize with extant cancer therapy. In the clinic McNeel *et al.* reported that combination anti-PD-1 immunotherapy with a T-cell stimulating DNA vaccine was well-tolerated and promoted antitumor responses [25]. A more recent study by Agarwall *et al.* tested the adaptive EP delivery of a synHPV vaccine for treatment of HPV positive head and neck cancers [26]. This phase Ib/II safety, tolerability, and immunogenicity study reported results of immunotherapy with MEDI0457 (DNA immunotherapy targeting HPV16/18 E6/E7 co-delivered with plasmid IL12) delivered by adaptive CELLECTRA-EP. Twenty-two patients with locally advanced, p16^+^head and neck squamous cell carcinoma (HNSCC) received MEDI0457. Overall the treatment was well tolerated. 90% of evaluable patients showed elevated antigen-specific T-cell activity by IFNγ ELISpot, and persistent cellular responses surpassing 100 spot-forming units (SFUs)/10^6^ peripheral blood mononuclear cells (PBMCs) were noted out to 1 year. Induction of HPV-specific CD8^+^ T cells was observed in tumors post biopsy. One patient developed metastatic disease and flow-cytometric analyses revealed induction of HPV16-specific PD-1^+^ CD8^+^ T cells that were not found before MEDI0547. Treatment of this patient with anti-PD-1 therapy resulted in a rapid and durable complete response. These data demonstrate that MEDI0457 can induce clear and durable HPV16/18 antigen-specific peripheral and tumor-infiltrating immune responses that may further benefit patients in the context of check point inhibitor (CPI) therapy to improve therapeutic outcomes. Interestingly, this group reported a second complete responder in the context of CPI combination therapy. Additional study of this approach is ongoing (ClinicalTrials.gov Identifier: NCT04001413). The combination of CPI with a potent T cell generating synDNA vaccine represents an important tool for additional study in the broader context of cancer immunotherapy.

## SynDNA biologics in the preclinical setting

The use of biologics and modified biologics to treat various diseases has rapidly expanded. Similarly, other immune disorders and cancers may be treated with monoclonal antibodies targeting cancer-specific antigens or immunosuppressive molecules present on immune cells such as cytotoxic T-lymphocyte-associated protein 4 (CTLA-4) and programmed cell death-1 (PD-1). Indeed anti-PD-1 and CTLA-4 immunotherapy have had success in the clinic and become standard of care cancer treatment approaches [27]. Recent reports have described use of the SynDNA-encoded monoclonal antibody (**DMAb**) therapy to deliver anti-PD-1 [28^••^] and anti-CTLA-4 [29^••^] immunotherapy *in vivo*. Bi-specific antibodies which engage cancer targets and cytotoxic T lymphocytes leading to T cell-mediated killing of cancer cells, are growing in clinical importance. The first FDA approved bispecific is Blincyto (Blinatumomab), which targets CD19 on B cells for treatment of acute lymphoblastic leukemia. Bispecfic drugs are complex to produce, can have short half-lives and as such, can be associated with high costs per treatment limiting their development and patient access. Taking into account its high local production in tissues, new SynDNA Ig production approach offer an alternative production pipeline for biologics. As an extension of this approach Perales-Puchalt reported on *in vivo* delivery with several months expression of a DNA encoded bispecific antibody delivered directly *in vivo* which demonstrated potent tumor control in a mouse model [30^••^] supporting the delivery of nonnative Ig forms.

Early reports described the use of plasmid vector systems for the generation of antibody like molecules as well as anti-HIV-1 envelope neutralizing F(ab)s [31]. Xu *et al.* reported the use of the synDNA platform to express and sulfate the antibody-like HIV-1 entry inhibitor eCD4-Ig [32^••^] illustrating the potential for complex *in vivo* biologic assembly. Recently, Wise *et al.* reported that DMAbs encoding HIV-specific broadly neutralizing antibodies could produce functional broadly neutralizing antibodies in mice and non-human primates [33^••^]. This platform continues to advance and DMAbs targeting influenza A and B [34,35], dengue [36], Chikungunya [37^••^], Zika [38^••^], and Ebola [35,39] with of protection in animal models.

DMAbs may also provide advantages as anti-bacterial therapeutics to complement antibiotics. Patel *et al.* reported that an optimized DMAb targeting the bacterium *Pseudomonas aeruginosa* protected against lethal pneumonia in a mouse model of antibiotic-resistant *P. aeruginosa* [40^••^]. Similarly, Wang *et al.* reported that a designed DMAb targeting the *Borrelia* protein OspA could protect against challenge with *Borrelia*-infected ticks, blocking Lyme disease transmission in the gut of the feeding ticks in a mouse challenge model [41]. These recent studies illustrate the growing interest in the DMAb approach. Additional study in this area is likely of high importance as a flexible and approach for prevention or treatment of infectious disease as well as for cancer therapy (Table 1) bringing many of the advantages of the simple DNA delivery platform.

**Table 1:** Selected preclinical studies for DNA-encoded biologics

Target	Disease etiology	Biologic class	Major findings	Ref.
Zika virus	Infectious	DMAb	Dual or single plasmid DNA delivery system results in expression of ZIKV neutralizing antibodies and serum from DMAb immunized mice, protects naïve mice from ZIKV lethal challenge.	[38^••^]
Ebola virus	Infectious	DMAb	EBOV DMAbs confer 100% protection from lethal challenge in mice/.	[35,39]
*P. Aeruginosa*	Infectious	DMAb	Anti-Pseudomonas DMAB protects mice from lethal pneumonia challenge and synergizes with antibiotic therapy resulting in protection from Antibiotic-resistant pneumonia.	[40^••^]
*B. burgdorferi*	Infectious	DMAb	Anti-Borrelia DMAb protects mice from tick challenge and represents a novel method for blocking Lyme disease transmission.	[41]
HIV-1	Infectious	F(ab)	Anti-HIV envelope neutralizing VRC01 F(ab) is produced rapidly *in vivo* following DNA immunization and EP.	[31]
HIV-1	Infectious	Broadly neutralizing Abs	Multiple bNAbs expressed in mice and NHPs at high concentration simultaneously, for extended periods	[33^••^]
Dengue	Infectious	DMAb	Delivery of multiple neutralizing DMAbs protects against all DENV serotypes and prevents antibody-dependent enhancement.	[36]
Chikungunya virus	Infectious	DMAb	A single injection of DMAb prophylaxis protects mice from CHIKV challenge. Combination of DNA and DMAb immunization affords both rapid and long-term protection.	[37^••^]
Influenza	Infectious	DMAb	DMAbs targeting influenza A and B protect mice from lethal challenge.	[34,35]
HIV-1	Infectious	Ig-like molecule	Proof-of-concept study for DNA-based delivery of anti-HIV immunoadhesins and *in vivo* modulation of protein function.	[32^••^]
PD-1	Malignancy	DMAb	Anti-PD-1 DMAbs are produced rapidly and persist in mouse sera, extending therapeutic window of immune checkpoint blockade therapy.	[28^••^]
CTLA-4	Malignancy	DMAb	Anti-CTLA-4 DMAbs are rapidly produced *in vivo* and shrink tumors in mouse cancer models.	[29^••^]
HER2	Malignancy	DMAb/DBiTE	Anti-HER2 DMAb and a bispecific targeting HER2 and CD3 induce control of ovarian tumors in mice and prolong survival.	[30^••^]

## Conclusions

The synthetic DNA platform has dramatically changed in its performance over the last 8 years. A combination of advances which have improved the delivery of the DNA into cells, increased tolerability, combined with multiple changes in genetic designs, and in formulations have resulted in a more potent vaccine platform with many of the features important for rapid vaccine development and deployment against emerging Infectious diseases. Later stage trials of these vaccines are worthy of significant attention. The extension of such studies to conserved non-viral cancer targets is similarly unique and important. Recent animal research studies showing disease impact from the biological delivery of monoclonal and bi-specific antibodies as DMAbs has primed an expansion of DNA-encoded biologics being investigated for infectious disease and cancer therapies in preclinical settings and has resulted in the first advance of DMAbs into this clinic (clinicaltrials.gov identifier: NCT03831503). The original pioneering DNA vaccine papers suggested the conceptual development and deployment advantages of a simple nucleic acid-based vaccine platform for impacting a host of global diseases [4^••^]. Recent advances in synthetic DNA technology have brought us steps closer to this important outcome and may have important implications for the development of vaccines against the newly emerged SARS-CoV-2.

## Conflict of interest statement

**D.B.W**. has grant funding, participates in industry collaborations, and has received speaking honoraria and fees for consulting. This service includes serving on scientific review committees and advisory boards. Remuneration includes direct payments or stocks/stock options and in the interest of disclosure, therefore, he notes potential conflicts associated with this work with, in particular, Inovio, where he serves on the BOD/SAB, as well as with Pfizer, Bristol-Myers Squibb, Merck, Aldevron, Roche, Ferring Pharmaceuticals, and possibly others.

## References and recommended reading

Papers of particular interest, published within the period of review, have been highlighted as

•• of outstanding interest
